# Targeted anticoagulant reversal, unintended consequences: lessons from andexanet alfa

**DOI:** 10.1093/ehjcvp/pvag014

**Published:** 2026-03-13

**Authors:** Jerrold H Levy, Joseph R Shaw

**Affiliations:** Departments of Anesthesiology, Critical Care, and Surgery, Duke University School of Medicine, 106 Research Drive, 2 Genome Court, MSRB 2, Room 1085, Durham, NC 27710, USA; Centre for Blood Research in the Life Sciences Center, University of British Columbia, Vancouver V6T 1Z1, Canada; Department of Medicine, University of Ottawa, Ottawa K1H 8L6, Canada; The Inflammation and Chronic Disease Program, Ottawa Hospital Research Institute, Ottawa, Ontario K1H 8L6, Canada

## The clinical need for anticoagulation reversal

Direct oral anticoagulants (DOACs) have transformed the prevention and treatment of thromboembolism, but like all anticoagulants, they require effective strategies to manage bleeding and to support urgent procedural or surgical interventions. Early criticism of DOACs focused on the lack of specific reversal agents. However, even for the vitamin K antagonists (VKAs), a four-factor prothrombin complex concentrate (4F-PCC) was not approved in the USA until 2013, despite its longstanding use elsewhere.^1^

Against this background, targeted reversal agents for DOACs were developed to address an important clinical need. Andexanet alfa (Andexanet), designed to reverse Factor Xa inhibitors (FXaIs), received accelerated Food and Drug Administration (FDA) approval in 2018 based on surrogate pharmacodynamic endpoints; however, subsequent data raised concerns regarding thromboembolic risk, and AstraZeneca voluntarily withdrew the drug from the US market in December 2025 following FDA review.^2^ This commentary explores the regulatory and clinical journey of andexanet from development to withdrawal.

## Development and mechanism

Andexanet was developed by Portola Pharmaceuticals as a reversal agent for FXaIs, with a particular focus on the long-acting FXaI betrixaban. However, because apixaban and rivaroxaban were approved earlier and rapidly achieved widespread clinical adoption, subsequent development focused primarily on their reversal. After an initial bolus and infusion, andexanet’s reversal effect lasts ~3–4 h; FXaI levels return to near baseline in apixaban-treated subjects, whereas in rivaroxaban-treated subjects, they rebound to levels exceeding those seen with placebo, an important consideration in clinical use.^3^

Andexanet is a catalytically inactive recombinant factor Xa molecule that functions as a competitive decoy receptor.^4^ By binding circulating FXaI, it restores endogenous factor Xa activity and re-establishes thrombin generation. Andexanet also binds and neutralizes tissue factor pathway inhibitor (TFPI), a key endogenous regulator of coagulation that inhibits the tissue factor–Factor VIIa extrinsic tenase complex, as well as early forms of the prothrombinase complex (FVa–FXa).^5^ Andexanet-mediated suppression of TFPI might increase thrombin generation and may induce a transient procoagulant state.^6^ This potential prothrombotic effect is indicated by a sustained increase in the endogenous thrombin potential (which measures total thrombin production), even after circulating FXaI levels rebound following the end of the andexanet infusion.^3^

## Initial think tank and Food and Drug Administration meeting

On 22 April 2014, a think tank meeting co-sponsored by the Cardiac Safety Research Consortium and the FDA was convened.^7^ The meeting brought together representatives from pharmaceutical companies, regulatory agencies, physicians, and scientists to address the lack of reversal agents for the DOACs and the development pathways required to advance such agents.^7^ Participants noted that anticoagulation for stroke prevention in atrial fibrillation (AF) remained underused, with the lack of a reliable reversal strategy frequently cited as a barrier. Although DOACs offered advantages in safety over VKAs even in the absence of reversal agents, there was broad consensus that specific reversal therapies would provide important clinical value, particularly in the setting of major bleeding. Given the impracticality of large outcome trials and the absence of suitable comparators, participants agreed that regulatory approval could reasonably rely on pharmacokinetic and pharmacodynamic data, preclinical safety, and the absence of adverse signals in early human studies.^7^ Bleeding management strategies were also discussed, including three reversal agents then in development: andexanet, idarucizumab, and ciraparantag.

## Andexanet: approval pathway and early evidence

Following initial volunteer studies demonstrating safety and pharmacodynamic efficacy, FDA approval of andexanet was supported by the ANNEXA-4 study, initially based on 352 patients,^8^ and the final report of 479 patients with major bleeding while receiving rivaroxaban or apixaban; 69% presented with intracranial haemorrhage (ICH) and 23% with gastrointestinal bleeding.^9^ The study was a single-arm, uncontrolled trial that measured the reduction in circulating FXaI levels as a surrogate endpoint. Haemostatic efficacy, as adjudicated by investigators, was rated as excellent or good in the majority of patients. Based on these surrogate endpoint data and the regulatory framework discussed at the 2014 FDA Think Tank, andexanet received accelerated approval in May 2018 for reversal of apixaban and rivaroxaban in patients with life-threatening or uncontrolled bleeding.

In the final ANNEXA-4 analysis, thrombotic events occurred in ∼10% of patients, although interpretation was limited by the lack of a comparator.^9^ Most patients had AF (81%), although vascular disease and recent thromboembolism were common. Accelerated approval had rested on rapid anti-Xa reversal in healthy volunteers and supportive single-arm data in bleeding patients, leaving unresolved whether biochemical reversal translated into improved haemostatic efficacy and clinical benefit relative to usual care. Those evidentiary limitations, together with the observed thrombotic signal and unresolved concerns about procoagulant effects related to TFPI suppression, led the FDA to require confirmatory randomized evidence. Because no approved active comparator existed in this setting, early discussions focused on a usual-care control framework rather than a labelled comparator.

## ANNEXA-I: randomized evidence and unwelcome clarity

Following the initial approval of andexanet, the FDA requested a randomized clinical trial comparing the agent with standard-of-care therapy. As a result, the ANNEXA-I trial evaluated patients with ICH, with 263 patients assigned to receive andexanet and 267 to usual care.^10^ Most patients had AF, and usual care predominantly consisted of 4F-PCC; among those in the usual-care group, 85.5% received 4F-PCC. Haemostatic efficacy was achieved in 150 of 224 patients (67.0%) treated with andexanet compared with 121 of 228 (53.1%) receiving usual care. However, thrombotic events occurred more frequently in the andexanet group (27/263; 10.3%) than in the usual-care group (15/267; 5.6%). There were no meaningful differences between groups in functional outcomes, as measured by the modified Rankin scale, or in 30-day mortality.

## Heparin resistance and cardiac surgery: an unanticipated complication

In patients undergoing cardiac surgery with cardiopulmonary bypass (CPB), clinicians began using andexanet off-label to reverse FXaI before or during urgent or emergent procedures. Subsequent reports described both profound heparin resistance and major thrombotic events during CPB.^11^ Mechanistically, andexanet may attenuate heparin’s anticoagulant effect by binding and neutralizing antithrombin in the presence of heparin. Chabata et al.^12^ showed that andexanet-associated heparin resistance prevents unfractionated heparin (UFH)–mediated prolongation of clotting time and suppression of thrombin generation; equilibrium modelling suggested that andexanet abolishes UFH dose responsiveness, producing an unpredictable anticoagulant dose–response relationship determined by circulating antithrombin and andexanet levels. Clinically, this is directly relevant during CPB, where failure to achieve appropriate anticoagulation based on activated clotting times (ACTs) may delay CPB initiation or precipitate circuit thrombosis.^12^ Mishima et al.^13^ conducted a review of 14 reported cases of andexanet-associated heparin resistance and found that the mean initial ACT was only 199 s despite additional heparin dosing (mean total, 1123 U/kg). Reservoir thrombosis occurred in 35.7% of cases, with two circuit exchanges.

## Economic, practical, and comparative considerations

Beyond efficacy and safety, the use of andexanet was associated with substantial cost and logistical challenges. Treatment costs were initially $25 000−$50 000 per patient, and administration required both an intravenous bolus and continuous infusion due to its short half-life. Many centres did not routinely stock the drug, and 4F-PCC emerged as a practical off-label alternative, supported by observational data, broad availability, lower cost, and ease of administration.

Importantly, in DOAC-treated patients with major haemorrhage, ongoing blood loss may be accompanied by declining anticoagulant concentrations and depletion of endogenous haemostatic factors. In this context, PCCs may support haemostasis by augmenting thrombin generation. As outlined in a prior review on the use of haemostatic therapy for DOAC-associated bleeding, PCCs should be viewed not as true reversal agents but as pro-haemostatic strategies to manage bleeding.^14^ Prothrombin complex concentrates and other factor concentrates are increasingly used therapeutically in perioperative haemorrhage and may also play a role when bleeding persists despite the use of reversal agents. In the setting of major haemorrhage, therapeutic priorities differ from those in less severe bleeding. Reversal of the anticoagulant removes one contributor to bleeding but, by itself, does not ensure haemostasis. Consequently, the use of reversal agents in actively bleeding patients should be integrated into a broader, multimodal haemostatic resuscitation strategy.^14,15^

## Implications for cardiologists

In AF, DOACs are prescribed by cardiologists, but major bleeding is rarely managed solely by cardiologists. Once patients are hospitalized, decisions about reversal, haemostatic resuscitation, urgent procedures, and anticoagulant re-initiation often involve emergency physicians, intensivists, haematologists, and, when surgery is required, anaesthesiologists and surgeons in addition to the prescribing cardiologist.

This highlights an important clinical reality: the clinicians who prescribe anticoagulants are often different from those who manage urgent bleeding complications. For cardiologists, the implication is practical. Reversal options differ across anticoagulants and should be understood, even if they are not the primary determinant of drug selection. Dabigatran has a specific approved reversal agent, idarucizumab, for emergency surgery or urgent procedures and for life-threatening or uncontrolled bleeding, with immediate reversal after administration. By contrast, with andexanet withdrawn from the market, management of FXaI-associated major bleeding now relies on pragmatic haemostatic strategies requiring close multi-disciplinary coordination. The andexanet experience underscores the importance of rigorous post-approval evaluation and the need for additional, evidence-based approaches to FXa inhibitor-associated bleeding.

## Data Availability

No new data were generated or analysed in support of this invited commentary.
